# Development and Experimental Testing of an Optical Micro-Spectroscopic Technique Incorporating True Line-Scan Excitation

**DOI:** 10.3390/ijms15010261

**Published:** 2013-12-27

**Authors:** Gabriel Biener, Michael R. Stoneman, Gheorghe Acbas, Jessica D. Holz, Marianna Orlova, Liudmila Komarova, Sergei Kuchin, Valerică Raicu

**Affiliations:** 1 Physics Department, University of Wisconsin–Milwaukee, Milwaukee, WI 53211, USA; E-Mails: biener@uwm.edu (G.B.); acbas@uwm.edu (G.A.); jdholz@uwm.edu (J.D.H.); komarova@uwm.edu (L.K.); 2 Aurora Spectral Technologies, Milwaukee, WI 53211, USA; E-Mail: mstoneman@auroraspectral.com; 3 Department of Biological Sciences, University of Wisconsin–Milwaukee, Milwaukee, WI 53201, USA; E-Mails: orlova@uwm.edu (M.O.); skuchin@uwm.edu (S.K.); 4 UWM-Small Businesses Collaboratory, University of Wisconsin–Milwaukee, Milwaukee, WI 53211, USA

**Keywords:** optical micro-spectroscopy, fluorescence, two-photon excitation, multi-photon excitation, energy transfer

## Abstract

Multiphoton micro-spectroscopy, employing diffraction optics and electron-multiplying CCD (EMCCD) cameras, is a suitable method for determining protein complex stoichiometry, quaternary structure, and spatial distribution in living cells using Förster resonance energy transfer (FRET) imaging. The method provides highly resolved spectra of molecules or molecular complexes at each image pixel, and it does so on a timescale shorter than that of molecular diffusion, which scrambles the spectral information. Acquisition of an entire spectrally resolved image, however, is slower than that of broad-bandwidth microscopes because it takes longer times to collect the same number of photons at each emission wavelength as in a broad bandwidth. Here, we demonstrate an optical micro-spectroscopic scheme that employs a laser beam shaped into a line to excite in parallel multiple sample voxels. The method presents dramatically increased sensitivity and/or acquisition speed and, at the same time, has excellent spatial and spectral resolution, similar to point-scan configurations. When applied to FRET imaging using an oligomeric FRET construct expressed in living cells and consisting of a FRET acceptor linked to three donors, the technique based on line-shaped excitation provides higher accuracy compared to the point-scan approach, and it reduces artifacts caused by photobleaching and other undesired photophysical effects.

## 1. Introduction

Spectral resolution of light emitted by intrinsic and extrinsic fluorescent probes (e.g., organic dyes, quantum dots, or fluorescent proteins attached to macromolecules of interest) [1–6], when used in conjunction with ultrafast lasers, combines the advantages of multi-photon microscopes (MPMs)—such as inherent image sectioning capability and increased discrimination between emission and excitation light [7–10]—with an ability to specifically identify molecules within the sample, based on their spectral fingerprint [5].

Among the various applications benefiting from spectral fingerprinting of molecules, of particular interest is Förster Resonance Energy Transfer (FRET) [11–13]. FRET relies on the use of a pair of fluorescent tags attached to the proteins of interest. When one of the tags (called a donor, D) is excited by light, the near field of its transition dipole induces another transition dipole into an unexcited nearby molecule (an acceptor, A), thereby transferring the excitation from D to A [11–13]. FRET has been used for detecting conformational changes in macromolecules [14–18], tracking complex formation [19], probing heteromerization of more than two proteins [20], analyzing chromatin compaction [21], and monitoring dynamic protein interactions [22]. More quantitatively, FRET has been used for determining the average size of protein complexes from average FRET efficiency values of populations of interacting molecules [23–25], as well as the average fraction of associated *versus* unassociated monomers in a population of homo-oligomerizing proteins [23,26]. Advances in the FRET stoichiometry theory [27,28], together with the advent of optical micro-spectroscopy technology [5] have led to the development of a FRET imaging method for the determination of the stoichiometry and relative disposition of the protomers (*i.e.*, quaternary structure) within a protein complex in living cells [5,29,30]; this method has been dubbed FRET spectrometry [31].

In spectrally resolved FRET, the donor and acceptor signals are separated from the combined measured signal using spectral decomposition methods. Most MPMs serially excite individual voxels in the sample by raster-scanning a diffraction-limited spot across the sample, using a pair of galvanometric scanners, and passing the emitted fluorescence back through the scanner (to descan it) before detection using a photomultiplier tube (PMT). In single-point-scan systems, spectral resolution is typically achieved through the use of a dispersive element, such as a prism or reflective grating, and projecting the spectrum onto an array of PMTs to resolve the fluorescence into several wavelength ranges. As the number of wavelengths is equal to the number of PMTs, spectral resolution in this arrangement is limited by the increased complexity and prohibitive costs associated with scaling up the number of detectors and associated electronics. In order to increase spectral resolution without increasing system complexity, a setup has been introduced which resolves the fluorescence into its spectral components by a transmission grating that projects light onto an electron-multiplying CCD (EMCCD) camera [5]. In this fashion, spectral resolution as high as 1 nm has been achieved. This resolution is necessary when more than two fluorescent species are present, such as when the sample itself also presents broad-spectrum autofluorescence that is hard to distinguish from the FRET signal itself. High spectral resolution is also needed in various hyperspectral imaging applications (see, e.g., [32] and references therein).

Unfortunately, the signal level in point-scanning single or multiphoton microscopes is reduced dramatically by the fact that only one voxel is excited at any given time, even when the beam is rapidly scanned along an entire line, which means that the sample at that voxel emits for only a fraction of the time it takes to scan the entire sample. When spectral resolution is added to such systems, the signal level decreases further due to the distribution of photons among multiple wavelength channels. To overcome this problem, one could increase either (a) the excitation power or (b) the scanning time. However, increasing excitation power leads to increased photobleaching or other undesired photophysical effects. Furthermore, in FRET studies, increasing the excitation power leads to simultaneous excitation of multiple donors within a protein complex, which causes the donors to compete for transferring the energy to the same acceptor. These effects can drastically alter the FRET efficiency data and result in an incorrect quaternary structure model for the protein under investigation. On the other hand, increasing the scanning time leads to motional blur in the image (due to molecular diffusion during the long scanning time) with consequent loss of information about the dynamics of the biological system of interest.

In this study, we describe a fluorescence micro-spectroscopic technique in which all sample voxels along a line are excited simultaneously (or in parallel), thereby achieving increased scanning and photon collection speed. The method relies on shaping the excitation beam into a line and using a camera to detect the signals emitted along the entire line. When integrated with the spectrally resolved MPM described previously [5], the method provided spatial resolution similar to that of the point-scan excitation modality [8], an acquisition speed comparable to that of commercial multiphoton and confocal microscopes that present no spectral resolution, and a sensitivity (*i.e.*, signal level for the same image acquisition time) that is more than two orders of magnitude higher than that of both our previous MPM [5] and other multiphoton or confocal microscopes with spectral resolution.

In order to assess the comparative performance of the point-scan and line-scan optical micro-spectroscopy methods, we used yeast cells (*S. cerevsisiae*) expressing a FRET construct composed of three donors (consisting of the green fluorescent protein GFP_2_ [33]) and one acceptor (yellow fluorescent protein, YFP [34]), with all four fluorescent proteins joined together via two-amino acids linkers. This construct, which represents an oligomer of higher order than a dimer and contains more than one donor, allowed us to reveal the presence of undesired photophysical effects that take place at high excitation powers such as those needed in to produce high enough signal level in the point-scanning method. This situation is highly relevant to many of the FRET studies aimed at protein complex stoichiometry and quaternary structure determinations [5,29,30,35]. The results of our FRET analysis indicated that the line-scan method presents increased accuracy and reduces artifacts caused by photobleaching and other photophysical effects, which often affect the point-scan method.

## 2. Results and Discussion

### 2.1. Brief Overview of the Line-Scan Approach

In order to achieve high acquisition speeds in two-photon excitation microscopy, some setups use multiple excitation beams arranged in a matrix [36,37]. This arrangement cannot be easily adapted to spectral resolution, as having a large number of beams along the vertical direction in the image (*i.e.*, along the spectral dimension) would lead to overlapping of the spectra originating from different focal spots in the sample. Other investigators proposed methods for rapid scanning of the excitation beam along a line in the sample [38,39], followed by detection of the fluorescence along the entire line using CCD cameras. While offering fast acquisition speed, the sensitivity of this method is still limited by the fact that, while the signal is collected from a certain sample voxel, the other voxels along an excitation line are not excited; hence, the parallel acquisition feature of the EMCCD detector is not fully taken advantage of in that photons are not detected simultaneously for all the pixels along a line in the image.

A more suitable way to excite several sample voxels simultaneously (or in parallel), and hence to increase scanning speed and detection efficiency, would be to shape the beam into a line and use a camera to detect the signals emitted along the entire line, as has been done previously [40]. This method is particularly effective when used in conjunction with spectral resolution, as acquisition of all the spectral components along an entire line in the sample is possible using an EMCCD camera. Unfortunately, some of the published setups relying on line scanning presented an axial point spread function (PSF) with full width half maximum (FWHM) of 5 μm (as measured using a thin fluorescent ink layer), which was lower (see [8], for a calibration) than that corresponding to a point-scan method, and it could only be slightly improved (~3 μm) by using a confocal slit [40].

In an attempt to re-evaluate the original line-scan method, we hypothesized that the reduction in the axial resolution presented by the earlier setups stemmed from the use of cylindrical lenses or spatial light modulators [40–43] (which present high dispersion of the first and higher order), which were generally used to replace the scanning lens in the conventional point-scan setup. In those schemes, (i) the first order dispersion introduces chromatic aberrations, which means that the broadband laser light is focused at different positions along the *z* axis, while (ii) the second order dispersion degrades the laser pulse properties by introducing chirp [44]. Both of these effects cause poor spatial and temporal focusing along the *z* axis and, hence, low axial resolution and reduced sensitivity. In addition, (iii) when used to replace the more common spherical scanning lens, a cylindrical lens acting on an initially round Gaussian beam generates a line along its longitudinal axis, which has non-uniform intensity and leads to non-uniform emission from the sample. The use of rather sophisticated methods, such as temporal focusing [41–43], improved the resolution (~1.5 mm) by increasing complexity and, hence, undesired sensitivity to optical alignment [41].

**Figure 1.**
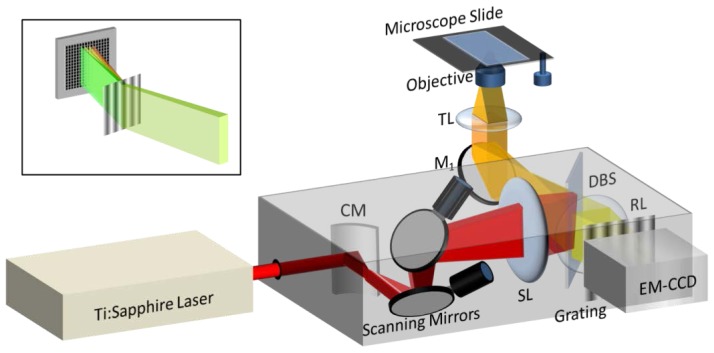
(Color online) Two photon excitation microscope with high spatial and spectral resolution using the line-scan excitation method. Significance of acronyms: CM, cylindrical mirror; SL, scanning lens; M_1_, plane mirror; TL, tube lens; DBS, Dichroic beam splitter; RL, imaging relay lens. Inset: Instantaneous spectrum measurement concept used by the two-photon excitation microscope in a line-scan configuration.

In this study, we have explored the use of a cylindrical mirror as a static “scanning device”, placed in the focal plane behind a spherical scanning lens (see Figure 1) of a two-photon microscope with spectral resolution. When adding this modification to the spectrally resolved MPM described previously [5] it produced an illumination PSF virtually identical to that of the point-scan excitation modality (described in [8]).

### 2.2. Design and Realization of the Optical Setups

A schematic of the two-photon microscope with line-shaped excitation beam and spectral resolution is shown in Figure 1. The near-IR beam from a Ti:Sapphire Laser (Mai Tai HP; Spectra Physics, Santa Clara, CA, USA) is directed to a cylindrical mirror which spreads the beam along one dimension (which we call *long axis* or *x*-axis) and leaves it unchanged along the perpendicular axis (called *short* or *y*-axis). The resulting beam impinges on a galvanometric mirror that scans it in the direction of the short axis. A spherical lens (*i.e.*, scanning lens), placed at the exit focal plane of the cylindrical mirror, focuses the beam in the direction of the short axis and collimates it along the long axis. The beam is then directed using a two-photon dichroic beam splitter (with a 650-nm cutoff wavelength), through a tube lens (200 mm focal length), and then focused by a 100× oil-immersion objective (NA = 1.45) from Nikon (Melville, NY, USA) onto the sample to a diffraction-limited spot. The fluorescent light emitted from the sample is collected by the same objective and tube lens, and imaged onto an EM-CCD (iXon3; Andor Technologies, South Windsor, CT, USA) using a 1× relay lens system with a focal length of 150 mm. Any residual back-scattered near-IR light that has passed through the dichroic mirror is filtered out using a short-pass filter (Chroma Technology, Bellows Falls, VT, USA) and an absorption filter that together provide light transmission of less than 10^−8^ for λ ≥ 650 nm. Before the fluorescence is detected by the camera, it is passed through a blazed Kinoform diffraction phase grating (300 grooves/mm, Thorlabs, Newton, NJ, USA) positioned about 53 mm away from the EMCCD chip, which disperses the light according to its spectrum in a direction (*y*) perpendicular to the excitation/emission line (see Figure 1 inset). This configuration allows for instantaneous detection of the entire spectrum for each image sample voxel with a spectral resolution of about 1.2 nm; the resolution may be changed by adjusting the distance between the grating and the camera.

The point-scan system utilizes exactly the same optics, electronics and mechanical components as the line-scan system, except that the cylindrical mirror is replaced by a flat mirror, which directs the beam into a pair of galvanometric mirrors (both of which are used to scan the beam) [5]. By contrast to the line-scan excitation system, the point-scan system excites a single point in the sample, and this excitation voxel is then raster scanned throughout the field of view using the two galvanometric mirrors.

To directly compare the performance of the point-scan system to that of the line-scan system, we have assembled the two systems side by side using the same microscope and excitation laser. A home-made point-scan scanning head [5] was coupled into the left-hand-side port of a Nikon Eclipse Ti-U inverted microscope (Nikon, Melville, NY, USA). The scanning head with line-shaped excitation beam, attached to the right-hand-side port of the same microscope, was a modified version of the commercial OptiMiS system (Aurora Spectral Technologies, Milwaukee, WI, USA) in which one of the flat mirrors was replaced by a circular mirror with a cylindrical surface (radius of curvature, 26 mm) purchased from Edmund Optics (Barrington, NJ, USA).

The optical components and settings of the scanning heads were identical, including the second galvanometric scanner, which is actually not necessary in the line-scan system. The EMCCD cameras also were identical (see above)—and therefore featured the same resolution (512 pixels × 512 pixels), pixels size (16 μm × 16 μm), quantum efficiency (~80%), *etc*., and the same settings were used for both of them (e.g., frame transfer rate, EM gain, *etc.*). As stated above, the only difference between the two set-ups was that a flat mirror in the point-scan set-up was replaced by a curved mirror.

The light from the Ti:Sapphire laser (with a spectrum centered around 800 nm) was split into two beams, one beam directed into the entrance aperture of the line-scan system, and the other directed into the entrance aperture of the point-scan system. The quality and shape of the beams after splitting remained the same as for the incident beam except perhaps for the slight (but undetermined) chirp that was expected to be introduced to the beam that was used for both systems after passing through ~1 cm of beam-splitting optics; in addition, the power was adjusted differently for the two beams as required by the excitation method used (see below). The two scanning heads, the microscope, the two EMCCD cameras (see above), and the laser where all controlled by the same computer using custom software written (in C++) in house for beam scanning, image acquisition, and reconstruction.

### 2.3. Characterization of the Point Spread Functions of the Line-Scan and Point-Scan Setups

In order to determine the point spread functions (PSF) of the two systems, we used fluorescence sources that could be considered point emitters, which is one of the most common methods for determining PSF in three dimensions in a variety of optical microscopy techniques [45–48]. As point-emitters, we used 0.175-μm orange fluorescent beads (SP Speck Microscope Point Source Kit, P7220, Molecular Probes, Life Technologies, Grand Island, NY, USA) deposited on the bottom of a glass-bottom dish filled with deionized water. In order to test the axial resolution, we have scanned the sample along the optical axis by changing the position of the sample stage relative to the objective in steps of 75 nm starting below the focal point and ending above it. The fluorescence intensity collected in each section was integrated over a whole area around a bead and plotted as a function of position of the stage. The lateral resolution (*i.e.*, in the *x* and *y* directions) was assessed by determining the PSFs in the focal plane of the microscope objective.

**Figure 2.**
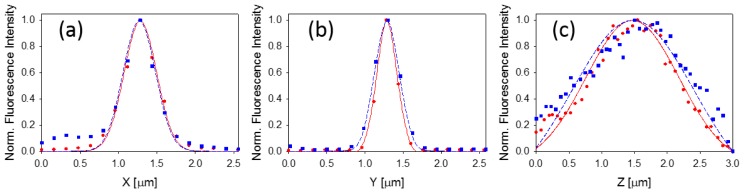
Point spread function (PSF) cross-sections along (**a**) *x*; (**b**) *y*; and (**c**) *z* axes as measured by the line-scan excitation (red circles) and the point-scan excitation (blue squares). The solid (red) and dashed (blue) lines represent Gaussian fits for the line-scan-and point-scan data, respectively. The resulting FWHM for *x*-PSF is 0.5 μm for both point-scan and line-scan. The resulting FWHM for *y*-PSF is 0.3 μm for line-scan and 0.37 μm for the point-scan. Finally, the resulting FWHM for *z*-PSF is 1.6 μm for line-scan and 2.0 μm for the point-scan.

As shown in Figure 2, the lateral PSF components took values between 0.3 and 0.5 μm in both systems, with a slight degradation in the *x* direction perhaps as a result of the asymmetry in the detection scheme (*i.e.*, detecting an entire line for each camera frame). The full-width half-maximum of the axial PSF was 1.6 μm for the line-scan and around 2.0 μm for the point-scan, which is comparable to the finest resolution reported for the more sophisticated line scan excitation methods without spectral resolution detection [40–42,49].

### 2.4. Comparison of Sensitivity and Acquisition Speed between the Two Scanning Modes

In order to directly compare the signal detection sensitivity (*i.e.*, signal level for the same image acquisition time) and the acquisition time of both the line- and the point-scan excitation systems, we performed measurements on an aqueous solution of Uranine (Fisher Scientific, Pittsburgh, PA, USA). About 2 mL of 100 μM Uranine solution was pipetted into a glass-bottom culture dish (MatTek Corporation, Ashland, MA, USA), which was mounted onto the stage of the Nikon Eclipse inverted microscope and held fix for the duration of all point-scan and line-scan measurements. The laser light was then focused just beyond the thin glass slab at the bottom of the dish, and then a rectangular area in the sample was scanned.

As the energy of the excitation beam was distributed in the sample differently between the two methods of excitation, we could not simply match the total laser powers delivered through the microscope objective in the two excitation methods. A good approximation is to match the excitation power delivered to a volume of the sample corresponding to a single pixel in the image space for the line scan, *P_pix,LS_*, to the power at the peak of the PSF falling across a distance corresponding to an image pixel in the point-scan, as we will describe next.

In the line-scan system, the excitation power is spread uniformly across the sample, *i.e.*, each excitation voxel along this line is only exposed to a fraction of the power that the objective is focusing onto the sample. In this case, *P_pix,LS_* *is simply given by:*

(1) $$P_{pix,LS}=\frac{P_{LS}}{n_{pixels}}$$

where *P_LS_* is the fraction of the measured power delivered by the objective to the sample adjusted for the same field of view as that of the point-scan, and *n_pixels_* is the number of pixels on the EM-CCD camera along which the emission line is spread.

To measure the excitation PSF (in the *x-y* plane) of the point-scan system, we scanned the laser beam across a 175-nm orange fluorescent bead (described above) in a series of steps, collecting the total emitted fluorescent signal at each step. The step size of the excitation voxel deflection corresponded to the distance the measured fluorescence traveled on the camera, which is equal to an image pixel size. We fitted this excitation PSF using a single Gaussian function,

$$PSF\;(x)=Ae^{\frac{-x^{2}}{2\sigma^{2}}}$$

(where *A* is a normalization factor) and computed the standard deviation of the Gaussian (σ) which corresponded to approximately 1.2 pixels. A geometrical approximation based on the graph of this Gaussian provided a simple estimate for the average (over multiple pulses in time) power per pixel corresponding to the peak of the PSF for the point scan,

(2) $$P_{pix,PS}=\frac{1}{\sigma \sqrt{\pi }}P_{PS}$$

where *P_PS_* is the total laser power passing through the microscope objective under point-scan conditions.

By setting the maximum IR power (*i.e.*, the rate of heating caused by the point scan to the sample) given by Equation (2) equal to the power in the line-scan configuration given by Equation (1), we obtain the following relationship between the total laser powers in the two scanning configurations:

(3) $$P_{LS}=\frac{n_{pixels}}{\sigma \sqrt{\pi }}P_{PS}.$$

Equation (3) was used as a reference in the sensitivity assays described below. Let us consider, for instance, an image line of 300 pixels and a point-scan power of 2 mW. This equation demands a power of 0.3 W for the line scan, not including the losses in the optical system (a factor of 3 or more) and the excess of power caused by the fact that the excitation line is longer by at least a factor of 3 than required by the field of view (so that the intensity distribution along the line is uniform). As a result, it is experimentally very difficult to capture the signals in both configurations with the camera when using the very different powers predicted by Equation (3), either because the point-scan power cannot be lowered enough and still achieve a good signal-to-noise ratio with the camera, or the laser cannot achieve the required power to match the line-scan to the point-scan. The net result of this is that the sensitivity achieved in the line-scan configuration will be inevitably underestimated.

The results of such sensitivity measurements are summarized in Table 1 for the case when *P_pix,PS_* was about 3.3 times higher than *P_pix,LS_*. In this set of measurements, first a point excitation scan and then a line excitation scan of the 100 μM Uranine solution was performed for a particular number of pixels scanned. The integration times for both configurations were set equal to the lowest value allowed by the frame rate of the camera (see caption to Table 1). Then, multiple point scans were performed for increasing values of the integration times until the signal detected upon a point-scan excitation (*W_PS_*) equaled that of the line-scan excitation (*W_LS_*). As evident from the results displayed in Table 1, an increase in the sensitivity or acquisition speed in the line-scan, compared to the point-scan acquisition, was detected, which was nearly linearly dependent on the total number of pixels scanned along a line in the image. Therefore, for a typical field of view (*i.e.*, Δ*x* > 300 pixels), both the increase in sensitivity and decrease in acquisition time obtained, by using the line-scan approach, exceeded two orders of magnitude.

### 2.5. Quantifying FRET in Biological Cells Using the Two Scanning Methods

As mentioned in the introduction, spectrally resolved FRET allows one to determine the binding stoichiometry and quaternary structure of proteins of interest in living cells using a single excitation scan of the sample. The highly resolved spectrum, measured for each pixel, is unmixed using a least-squares minimization procedure described previously [5,50]. Briefly, the method considers the overall spectrum as a linear combination of elementary spectra of donors and acceptors, and determines the proportionality constants multiplying each elementary spectrum by minimizing the sum of the squares of the point-by-point difference between the simulated and measured spectra. In contrast to conventional average intensity-based methods, this method relies on the analysis of distributions of apparent FRET efficiencies, *E_app_*, across the image pixels of individual cells expressing proteins of interest. The most probable quaternary structure of the complex is identified from the number of peaks in the distribution of FRET efficiencies and their mutual relationships. Such peaks collectively create a unique FRET fingerprint of a specific oligomer (quaternary) structure [31,50], or a “FRET spectrum” of the complex. The theoretical modeling used to determine the quaternary structure assumes that (i) no significant photo-bleaching of the fluorescent tags occurs while scanning the sample, and (ii) only one donor in a complex containing more than two fluorescent tags is excited for every laser pulse (that is, a donor does not compete with other donors to transfer energy to an acceptor). If either of these assumptions is not fulfilled, the models become extremely complicated or even intractable. Given these effects, the point-scan method faces the conflicting requirements that the excitation intensity is low enough to avoid the above effects, while, on the other hand, the power is high enough to produce high signal levels. For the sake of completeness, it should be noted that an additional assumption of the theoretical models used to interpret FRET spectra (which, however, has no bearing on the present comparison between line-scan and point-scan excitations) is that (iii) the FRET efficiencies at each pixel originates from a single donor acceptor configuration in a molecular complex. This is practically achieved if either the expression level is low (*i.e.*, there is on average less than one complex per pixel), or, at high expression levels, the different oligomeric configurations are spatially separated.

**Figure 3.**
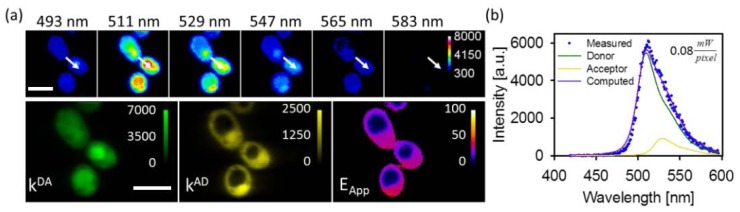
Typical results obtained from yeast cells expressing a FRET construct (DDDA) as measured with the line-scan optical micro-spectroscopy systems. Images in the top row of panel (**a**) illustrate the fluorescence emission images of the cells acquired at different wavelengths selected from a total of 200 wavelengths over the 420–600 nm range. These were unmixed into donor emission in the presence of acceptor (*k^DA^*) and acceptor emission in the presence of donor (*k^AD^*), from which the apparent FRET efficiency distribution (*E_App_*) was computed for each image pixel (see the Methods section); and (**b**) Fluorescence spectra corresponding to the pixel indicated by the white arrows in (**a**). Measured fluorescence spectra are represented by filled circles, unmixed donor spectrum by green solid line, unmixed acceptor spectrum by yellow solid line, and the best-fit simulated spectrum by purple solid line. Scale bars, 5 μm.

In order to test the points (i) and (ii) raised above, we used yeast cells expressing a FRET construct composed of three donors (consisting of GFP_2_, [33]) and one acceptor (YFP [34]) with all modules joined together via a two-amino acid linker (see the Methods section). We denoted this construct “DDDA”, which symbolizes three donors serially linked to each other and then linked to one acceptor. We chose this construct, firstly, because it represents an oligomer that is higher than a dimer and, secondly, because it contains more than one donor, which allows for donor competition to take place at high excitation rates. This situation is highly relevant to many of the FRET studies aimed at protein complex stoichiometry and quaternary structure determinations [5,29,30,35]. Figure 3 presents a typical FRET result obtained with the line-scan method. The average excitation power was 0.08 mW/pixel, while the integration time was 50 ms. As one may readily see, there are some differences between the *k^DA^* and *k^AD^* maps in Figure 3 in that certain regions where the donor signal is rather high are devoid of acceptor signal. This resulted in a non-uniform FRET efficiency throughout the cell. We attributed these differences to differences in the maturity of the donor and acceptor molecules in the tetrameric constructs. Specifically, since GFP_2_ was designed to fold comparatively faster, it becomes fluorescent sooner after the synthesis compared to YFP. The differences in donor and acceptor signal levels are therefore more obvious in the endoplasmic reticulum where these constructs are synthesized.

In order to test photobleaching and other photophysical effects we have repeatedly scanned cells with the same excitation power. The point-scan excitation power was chosen based on the highest signal level achievable without severely photobleaching the sample, yet always remained under that used in the line scan system. The measurements were repeated 10 times for each field of view while keeping the exposure time constant from frame to frame and the same for both methods. The analysis of the results included calculation of the FRET efficiency map as described above and extraction of the FRET efficiency histogram for individual cells.

**Figure 4.**
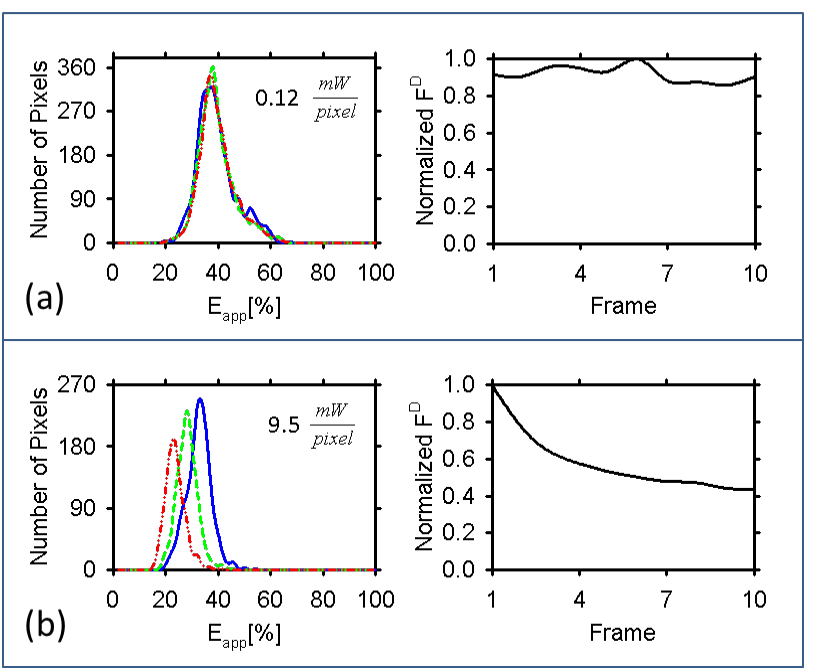
*E_app_* histograms of a cell calculated from the FRET efficiency map as measured using the line-scan system (**a**) and the point scan system (**b**) after repeated scans of the sample along with the normalized emission of the donors corrected for FRET. Pixels for which either *k^DA^* or *k^AD^* values were within less than 2 standard deviations of the noise level were discarded from the analysis. Blue solid line: scan 1; green dashed line: scan 4; red dashed-dotted line, scan 7. The maximum values for the *F*^D^ were 38,400 and 14,775 for (**a**) and (**b**) respectively, for these cells.

Figure 4 summarizes the results from that experiment. As can be seen, for the line-scan excitation method there was no obvious change in the FRET efficiency histogram or the emission intensity throughout the measurements. By contrast, in the point-scan excitation method, the change in the FRET efficiency histograms from scan to scan was obvious, and it was accompanied by a definite reduction in emission intensity. More specifically, for the line-scan excitation scheme the intensity fluctuated in the range of ±7% of the initial intensity, while the FRET efficiency histograms did not apparently change after repeated scans. When using the point-scan excitation scheme, on the other hand, we noticed a reduction in the measured donor intensity throughout the experiment, down to 40%–45% of the initial measured intensity. What is worse, the histograms changed markedly between measurements, in both their height and their peak position.

The decrease in the histogram height could be easily correlated with donor photobleaching: that is, fewer emitting molecules result in fewer pixels showing signal above noise level and hence FRET; note that pixels presenting low signal to noise were excluded from the analysis (see caption to Figure 4). By contrast, the leftward shift in the peak position could not be ascribed to donor photobleaching alone, as this would normally lead to an increase in the FRET efficiency (which is inversely proportional to the number of donors in a complex) [5], *i.e.*, a peak shift to the right. This suggests that the acceptor too might have been photobleached, which would generate DDD complexes that would mix their signal with DDDA complexes and therefore lead to a reduction in the apparent FRET efficiency measured.

Other effects that could be at play include competition between the quasi-simultaneously excited donors for transferring energy to the unexcited acceptor, and singlet-singlet annihilation processes [51]. Both of these effects, which require the presence of more than one donor of energy in the complex, may become significant at high excitation powers, as might easily be the case with the point-scan measurements described in this paper. They could lead to an effective decrease in the donor emission and hence an apparent reduction in FRET efficiency.

For the DDDA FRET construct used in this study, we conclude that the shift in FRET efficiency is perhaps affected by several possible effects discussed above, which are all power-dependent. Having performed several measurements, we noticed that, for the point-scan excitation the peak position of the FRET efficiency histograms occasionally shifted to the opposite (*i.e.*, increasing) direction. This was perhaps due to changes in the relative contributions of the various photophysical effects from cell to cell. Fortunately, the low excitation powers needed in the line-scan approach provide one with the means to avoid such effects rather than account for them theoretically, which is no easy task. Thus, the line-scan excitation method is better suited to biological imaging, both because of its higher accuracy and sensitivity (or speed).

## 3. Methods

Yeast cells (*S. cerevisiae*) were genetically engineered to express a FRET construct composed of three donors (consisting of GFP_2_, [33]) and one acceptor (YFP [34]) with all modules joined together via a two amino acid linker (Ser-Gly). This construct was built using molecular genetic methods by stepwise modification of a GFP_2_-Ser-Gly-YFP construct described previously [5]. The resulting plasmids were inserted into the cells using standard methods [5]. The cells were imaged using the two set-ups described above and the resulting images were unmixed using a least-squares minimization algorithm into donor emission in the presence of acceptor (*k^DA^*) and acceptor emission in the presence of donor (*k^AD^*). From these, the apparent FRET efficiency distribution (*E_App_*) was computed for each image pixel using the equation *E_app_* = [1 + (*k^DA^Q^A^w^D^*)/(*k^AD^Q^D^w^A^*)]^−1^, where *Q^D^* (*Q^A^*) and *w^D^* (*w^A^*) are the quantum yield and the spectral integral of the donor (acceptor), respectively [5,50].

## 4. Conclusions

We have built, side by side, a point-scan and a novel line-scan two-photon optical micro-spectroscope, in order to assess comparatively their performance. The line-scan system presented an increased sensitivity and/or acquisition speed, compared to the point-scan system, while the spatial resolution was comparable for the two systems. For a field of view of 300 pixels in the direction of the excitation line, both the increase in sensitivity and decrease in acquisition time presented by the line-scan approach exceeded two orders of magnitude. For larger fields of view, the differences are expected to be even larger, since the integration time for the point-scan excitation increases with the number of pixels, while for the line-scan it does not. In order to achieve comparable performance with the two systems, in terms of sensitivity and acquisition speed, we attempted to dramatically increase the excitation power per pixel for the point-scan set-up and then compared the results obtained with both systems from measurements of a fusion construct of fluorescent proteins that was expressed in yeast cells as a tetrameric FRET standard. The increase in excitation power in the point-scan system not only led to rapid destruction of the fluorescent proteins, as revealed by a rapid decrease in fluorescence intensity, but also provided inconsistent FRET efficiencies from scan to scan. We therefore conclude that the line-scan system here introduced achieves superior performance to that of the point-scan micro-spectroscope previously introduced by us and may eventually lead to an ability to probe changes in protein-protein interactions in living cells with high accuracy.

## Figures and Tables

**Table 1.** Comparison of the relative signal level between the point- and line-scan (*W_PS_* and *W_LS_*), respectively, when scanning a solution of 100 μM Uranine. The average power per pixel for line-scan was 0.72 mW and the average power per pixel measured at the peak of the PSF for the point-scan was 2.37 mW. The line-scan was executed using the lowest line dwell time allowed by the frame rate of the EMCCD (Δ*t*_LS_ = 29 ms for 300 pixels and 150-pixel lines and 33 ms for 512-pixel lines).

Δ*x*(*pixels*)	$\frac{\Delta t_{PS}}{\Delta t_{LS}}$	**$\frac{W_{LS}}{W_{PS}}$**
150	1.0	96.0
150	103.0	1.0
300	1.0	186
300	192.0	1.0
512	1.0	280
